# Genome-Wide Evidence for Complex Hybridization and Demographic History in a Group of *Cycas* From China

**DOI:** 10.3389/fgene.2021.717200

**Published:** 2021-08-30

**Authors:** Yueqi Tao, Bin Chen, Ming Kang, Yongbo Liu, Jing Wang

**Affiliations:** ^1^Key Laboratory of Plant Resources Conservation and Sustainable Utilization, South China Botanical Garden, Chinese Academy of Sciences, Guangzhou, China; ^2^University of Chinese Academy of Sciences, Beijing, China; ^3^Shanghai Chenshan Botanical Garden, Shanghai, China; ^4^Eastern China Conservation Center for Wild Endangered Plant Resources, Shanghai, China; ^5^Center of Conservation Biology, Core Botanical Gardens, Chinese Academy of Sciences, Guangzhou, China; ^6^State Environment Protection Key Laboratory of Regional Ecological Process and Functional Assessment, Chinese Research Academy of Environmental Sciences, Beijing, China

**Keywords:** conservation genomics, *Cycas*, demographic history, gene flow, RADseq

## Abstract

Cycads represent one of the most ancestral living seed plants as well as one of the most threatened plant groups in the world. South China is a major center and potential origin of *Cycas*, the most rapidly diversified lineage of cycads. However, genomic-wide diversity of *Cycas* remains poorly understood due to the challenge of generating genomic markers associated with their inherent large genomes. Here, we perform a comprehensive conservation genomic study based on restriction-site associated DNA sequencing (RADseq) data in six representative species of *Cycas* in South China. Consistently low genetic diversity and strong genetic differentiation were detected across species. Both phylogenetic inference and genetic structure analysis via several methods revealed generally congruent groups among the six *Cycas* species. The analysis with ADMIXTURE showed low mixing of genetic composition among species, while individuals of *C. dolichophylla* exhibited substantial genetic admixture with *C. bifida*, *C. changjiangensis*, and *C. balansae*. Furthermore, the results from Treemix, *f*_4_-statistic, and ABBA-BABA test were generally consistent and revealed the complex patterns of interspecific gene flow. Relatively strong signals of hybridization were detected between *C. dolichophylla* and *C. szechuanensis*, and the ancestor of *C. taiwaniana* and *C. changjiangensis*. Distinct patterns of demographic history were inferred for these species by Stairway Plot, and our results suggested that both climate fluctuation and frequent geological activities during the late Pleistocene exerted deep impacts on the population dynamics of these species in South China. Finally, we explore the practical implications of our findings for the development of conservation strategies in *Cycas*. The present study demonstrates the efficiency of RADseq for conservation genomic studies on non-model species with large and complex genomes. Given the great significance of cycads as a radical transition in the evolution of plant biodiversity, our study provides important insights into the mechanisms of diversification in such recently radiated living fossil taxa.

## Introduction

Cycads (Cycadales) represent one of the oldest living seed plants. They originated about 300 million years ago (Mya) in the Permian and reached dominance during the Jurassic–Cretaceous period (Mamay, 1969). However, extant cycad species originated within the past 12 million years and have been identified as a kind of evolutionary relict (Nagalingum et al., 2011). Cycads now comprise two families (Cycadaceae and Zamiaceae) and ten genera, with all species restricted to tropical and subtropical areas (Calonje et al., 2020). They exhibit intermediate morphological features between less-evolved plants (such as ferns) and more-advanced plants (including the angiosperms), e.g., with motile gametes and circinate vernation being the plesiomorphic traits, and the production of pollen and seed as the apomorphic characters, making them an ideal research system for plant evolution (Brenner et al., 2003). Over the past several decades, cycads have experienced global decline due to climate change, habitat degradation, and overexploitation for their great economic value (Norstog and Nicholls, 1997), leading to 62% of species being classified as threatened or even extinct (International Union for Conservation of Nature, 2020). *Cycas* L. is the only member currently known in the Cycadaceae, and it is the largest genus of the extant cycads, with a total of 118 species (Condamine et al., 2015). South China is a major center and potential origin of *Cycas* (Xiao and Möller, 2015), harboring around 25 species (Calonje et al., 2020). Despite the vast species richness, nearly all of the species in China are found with extremely small populations in the wild. Consequently, all living *Cycas* species have been listed in the Appendix of the Convention on International Trade in Endangered Species.

Genetic diversity is important for plant species to persist in the face of threats to their survival (Frankham, 2005). Populations harboring low genetic diversity are expected to have a reduced capacity to cope with environmental changes, because genetic diversity partially reflects their demographic history, and part of the neutral genetic diversity involves hitchhiking with adaptive genetic variation (Teixeira and Huber, 2021). Small populations tend to have lower genetic diversity than large ones, and rare species have significantly lower genetic diversity than their common counterparts, which was clearly demonstrated in a comparative survey of 247 plant species at a generic level (Cole, 2003). Furthermore, species with small populations may suffer from an increase in inbreeding and accumulation of deleterious mutations, which can further reduce their adaptive potential and dramatically increase the risk of extinction (Frankham, 2003, 2005; Heller and Zavaleta, 2009). Therefore, a comprehensive understanding of genetic diversity and demographic history is necessary for developing and implementing effective conservation strategies (Frankham, 2005; Feng et al., 2014; Teixeira and Huber, 2021). However, despite considerable efforts to understand genetic information of *Cycas* species in China (e.g., Xiao and Gong, 2006; Gong et al., 2015; Yang et al., 2016; Feng X. et al., 2017; Xiao et al., 2020), little is known about genomic-level genetic variation and demographic history in such taxa, given that all of the previous studies merely rely upon a few molecular markers covering a very limited subset of the genome of *Cycas* (up to 30 giga base pairs; Roodt et al., 2017).

The dramatic developments in next-generation sequencing technologies have enhanced the accessibility and availability of genomic resources at an unprecedented rate, thus providing a great opportunity to overcome the long-term limitations for the genomic study of non-model organisms. However, for organisms with large genome sizes, genome assembly remains a significant challenge in gaining reliable full genome resources; thus, reduced-representation library sequencing is considered to be the most promising technical alternative for such taxa (Clugston et al., 2019; Gargiulo et al., 2021). Restriction-site associated DNA sequencing (RADseq), combining enzymatic fragmentation of genomic DNA with high-throughput sequencing, generates large numbers of markers at a low cost (Baird et al., 2008). RADseq has been increasingly employed in conservation genetics in the last two decades (Wright et al., 2020), owing to the advantage that it does not require any prior genomic knowledge (Andrews and Luikart, 2014). The strengths of RADseq make answers to questions that were intractable using traditional markers (e.g., allozymes and microsatellites) more easily accessible, thus facilitating the transition of conservation genetics to conservation genomics that is involved in several study areas (Ouborg et al., 2010; Steiner et al., 2012), including phylogenetic reconstruction (Sovic et al., 2016), genetic diversity (Hendricks et al., 2017), population structure (Andrews et al., 2018; Rengefors et al., 2021), and demographic history (Dierickx et al., 2015).

*Cycas* was divided into six sections according to reproductive organs, namely, section *Asiorientales*, sect. *Stangerioides*, sect. *Indosinenses*, sect. *Cycas*, sect. *Panzhihuaenses*, and sect. *Wadeae* (Hill, 1994, 2008; Lindstrom et al., 2008; Liu et al., 2018). Specifically, there are 18 species occurring in China within section *Stangerioides* (Zheng et al., 2017), which is a polyphyletic group encompassing a majority of *Cycas* species scattered in south and southwest China (Hill et al., 2004). In the present study, six representative species with typically small populations, namely, *C. bifida* K. D. Hill (Hill, 2008), *C. changjiangensis* N. Liu (Liu, 1998), *C. dolichophylla* K. D. Hill, T. H. Nguyen, and K. L. Phan (Hill et al., 2004), *C. szechuanensis* W. C. Cheng and L. K. Fu (Cheng et al., 1975), *C. taiwaniana* Carruth. (Carruthers, 1893), and *C. balansae* O. Warburg (Warburg, 1900) were selected as a case study to estimate the genetic variation, interspecific gene flow and demographic history of such taxa with great conservation value. *C*. *dolichophylla* occurs mainly in the Red River region (Fragnière et al., 2015), while *C. changjiangensis* is endemic to the Bawangling National Nature Reserve in Hainan Province, China (Chen and Liu, 2004). *C. bifida* and *C. balansae* are mainly distributed in Guangxi and Yunnan Provinces, China (Hill, 2008). For *C. taiwaniana*, most extant individuals are cultivated in Fujian Province in China whereas they are rare in the wild (Hill, 2008). *C. szechuanensis* currently has only about 300 cultivated individuals in Guangdong, Fujian, and Sichuan provinces, with no wild populations recorded (Zheng et al., 2017). Therefore, all these species have been listed in the widely accepted concept of “Plant Species with Extremely Small Populations” (Wade et al., 2016), with the exception of *C. balansae*.

In this study, we utilized RADseq to perform comprehensive conservation genomic analyses in the six *Cycas* species. The specific goals of this study were to: (i) investigate the levels of genome-wide genetic diversity across species, (ii) determine the evolutionary relationship and characterize the genetic structure among species, and (iii) uncover the patterns of interspecific gene flow and demographic history. Finally, we discuss the practical implications of our findings for developing conservation strategies.

## Materials and Methods

### Sample Collection, DNA Extraction and Library Preparation

**FIGURE 1:**
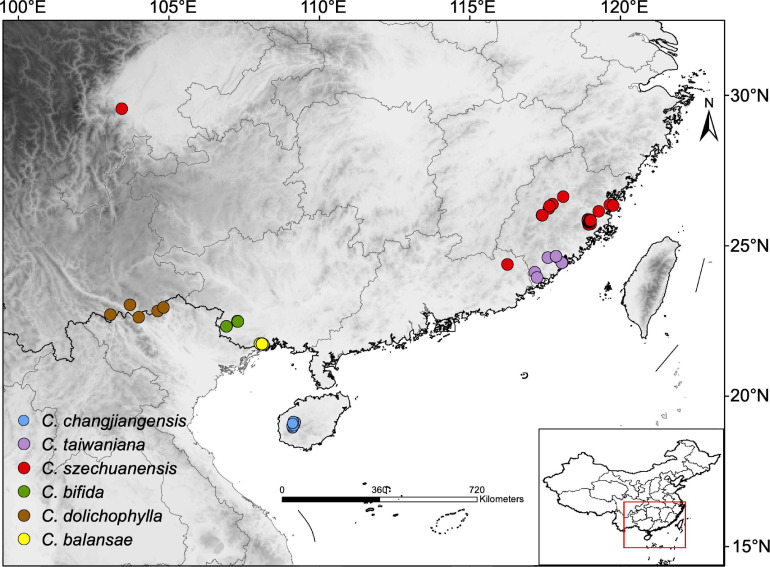
Map of sampling sites (inset map shows the sampling location on a map of China) for the six species of *Cycas* analyzed in the present study. Each dot represents a sampled individual and individuals are colored by species.

A total of 152 samples from 34 populations (one to 21 individuals per population) of the six *Cycas* species were collected: *C. bifida* (*n* = 18), *C. changjiangensis* (*n* = 52), *C. balansae* (31), *C. taiwaniana* (9), *C. szechuanensis* (*n* = 37), and *C. dolichophylla* (*n* = 5; Supplementary Table 1 and Figure 1). Leaf tissues were stored in silica gel during the field investigation. DNA for each sample was extracted from silica-dried leaves using a modified cetyl trimethylammonium bromide protocol (Doyle and Doyle, 1987). An average of 8 Gb reads per sample was captured by pair-end (PE) 150 bp RAD sequencing with restriction enzyme *EcoR1* and completed on a NovaSeq 6000 platform at Novogene Bioinformatics Institute (Beijing, China). Raw paired-end sequence reads were then subjected to a succession of filtering steps (Feng C. et al., 2017). To retain only the loci with high quality data for downstream analyses, reads with a minimum quality score of 20 (q20) below 90% were filtered out.

### SNP Calling

The pipeline STACKS v2.41 (Rochette et al., 2019) was used to generate single-nucleotide polymorphism (SNP) datasets. Given that no reference genome was available, and a large set of sequencing data was used in this study, it was necessary to optimize *de novo* assembly parameters before the final SNP calling. A series of parameter combinations were tested using some representative samples to identify the one that maximized the number of SNPs and minimized the genotyping errors. Consequently, the parameter set “m3M2n3,” which obtained the most SNPs (Supplementary Table 2) and exhibited relatively stable preliminary principle component analysis (PCA) results, was selected (Supplementary Figure 1). In detail, a stack could be created in ustacks with at least three depths of coverage; at the same time, a maximum of two mismatches were allowed between putative alleles when merging a locus, and a maximum of three mismatches were allowed between putative loci in cstacks when constructing a catalog. Three samples for each species (18 individuals in total) were randomly chosen to generate a catalog with cstacks, as this program is computationally too demanding to analyze such a large volume of sequencing data. Subsequently, all of the individuals were matched against the catalog in sstacks. Finally, in the populations program, loci that occurred in at least five species and in at least 70% of individuals per species were retained to reduce the missing rate. The minimum minor allele frequency (MAF) was set at 0.02 to process a nucleotide site at a locus. Further, sites with a maximum observed heterozygosity of 0.5 were kept to preclude paralogous sequences. Loci with exceptionally high coverage (i.e., larger than twofold standard deviations above the mean) were removed using vcftools v0.1.13 (Danecek et al., 2011). Input files for downstream analyses were converted with PGDSPIDER v2.1.1.5 (Lischer and Excoffier, 2012).

### Genetic Diversity

Species-level genetic statistics, namely, the inbreeding coefficient (*F*_IS_), observed heterozygosity (*H*_O_), and expected heterozygosity (*H*_E_) were computed at variable sites, as well as nucleotide diversity (π) at both variants and fixed positions in STACKS. Individual heterozygosity was calculated in STACKS to measure the extent of inbreeding. Furthermore, to quantify the degree of genetic similarity among individuals, pairwise kinship coefficients were estimated with NgsRelate (Korneliussen and Moltke, 2015). Pairwise genetic differentiation (*F*_ST_) among species was calculated with the populations program implemented in STACKS. Additionally, pairwise sequence divergence (*D*_XY_) among species was calculated with DnaSP v6.12.03 (Rozas et al., 2017), as the use of absolute measures of divergence is especially necessary when comparing species with different levels of inbreeding (Charlesworth, 1998).

### Phylogenetic Inference

We first used SVDquartets algorithm (Chifman and Kubatko, 2014) implemented in PAUP v4.0 (Swofford, 2003) with 100 bootstrap replicates to infer the species-level phylogenetic relationship for the six *Cycas* species by sampling 100,000 quartets. This approach was well suited for short gene sequences from the RADseq dataset, as it used each SNP occurring in the four-taxa alignment to maximize phylogenetic information (Eaton et al., 2017). The results were visualized in FigTree v1.4.4. We also reconstructed a maximum likelihood (ML) tree using IQ-Tree v1.7 (Nguyen et al., 2015). The best-fit model of TVM + F + R3 was selected according to Bayesian Information Criterion model selection (Kalyaanamoorthy et al., 2017). The model of TVM + F + R3 was applied, together with ascertainment bias correction (i.e., +ASC model), as the standard nucleotide substitution models do not incorporate the fact that only variable sites are included in SNP dataset (Lewis, 2001). Subsequently, 1,000 bootstrap replicates were used for UFBoot (Hoang et al., 2017) to calculate their support values. For both analyses, *C. balansae* was set as the outgroup based on its relatively basal status detected in a previous phylogenetic study of *Cycas* (Liu et al., 2018).

### Genetic Structure

The genetic structure among these species was examined using three different approaches. First, a Bayesian clustering method was performed using ADMIXTURE v1.3.0 (Alexander et al., 2009) to assign individuals to pre-defined clusters (denoted by *K*) according to their genotypes. We converted the variant call format file to browser extensible data format with Plink v1.90 (Chang et al., 2015). Independent runs were performed for each value of *K* from one to nine. The *K*-value with the smallest value of cross-validation error was selected as the optimal number of ancestral ingredients. Second, PCA, a dimensionality-reduction method without a prior assumption, was conducted to further assess the population structure with Plink. Third, a discriminant analysis of principal components (DAPC) was performed with the R package “adegenet” (Jombart, 2008) to further display population clustering, which could maximize between-group variation (Jombart et al., 2010). Finally, the results were visualized in R v3.6.2 (R Core Team, 2019).

### Interspecific Gene Flow

We first used a composite-likelihood approach implemented in Treemix v1.13 (Pickrell and Pritchard, 2012) to test for gene flow among the six species. The Treemix algorithm was run for up to five migration events using the –m parameter. The root was defined using the –root parameter, considering *C. balansae* as an “outgroup” in a phylogenetic context. Residuals were used to choose the best-fit model. Second, we calculated the *f*_4_-statistic (Reich et al., 2009), a powerful measure to discriminate between gene flow and incomplete lineage sorting (ILS), based on the unrooted population topology (A,B),(C,D). *f*_4_-statistic has been demonstrated to be effective in detecting introgression, regardless of whether the taxa tested are distant or sister species (Reich et al., 2009; Martin et al., 2015; Meyer et al., 2017). The *f*_4_-statistic is expected to be zero in the absence of introgression, no matter whether ILS is present or not. The traditional use of jackknife standard errors for calculating confidence interval (CI) presumes that the underlying data follow a normal distribution, which may often not be the case for the *f*_4_-statistic, especially in studies across highly divergent species (Meyer et al., 2017). Hence, we calculated the *f*_4_-statistic with the software F4, in which a novel simulation-based approach was developed to assess significance using a wrapper script for fastsimcoal2 (Meyer et al., 2017). Briefly, fastsimcoal2 was used to produce simulated sequence datasets similar to the true dataset in terms of size and amount of missing data, after which 1,000 sets of coalescent simulations were performed in a given four-species comparison. The resulted *f*_4_-statistic was considered as evidence for introgression if only less than 5% of the simulated datasets without introgression produced *f*_4_ values as extreme as the observed dataset. Additionally, ABBA-BABA analysis (*D*-statistic) was conducted with Dsuite v0.3 (Malinsky et al., 2021). The ABBA-BABA test was performed on a four-taxon tree in the form (((P1, P2), P3), O), where P1 and P2 are recognized to be closely related, P3 is a third ingroup taxa and O represents an outgroup. A significant deviation of *D*-statistics from zero due to different numbers of shared sites between P3 and either P1 or P2 indicates the occurrence of gene flow. ABBA represents that P2 and P3 have more shared alleles while BABA means that P1 and P3 share a greater number of alleles. The phylogenetic tree file was provided for reference before running Dsuite to generate combinations representing that P1 and P2 were more closely related. A subset lineage of *C. balansae* was used as the outgroup according to the phylogenetic tree.

### Change in Population Size

Stairway Plot v2 (Liu and Fu, 2020) was used to infer temporal changes in the population size (*N*_*e*_) for each species. A common mutation rate of 1.0 × 10^–8^ per site per generation was used following Liu and Hansen (2017) due to the lack of a precise SNP mutation rate reported for *Cycas*, and 40 years was set as a generation (International Union for Conservation of Nature, 2020) in the present study. All of the samples for *C. bifida*, *C. taiwaniana*, and *C. dolichophylla* were kept due to their small sample size, whereas we downsampled 20 individuals per species for *C. changjiangensis*, *C. balansae*, and *C. szechuanensis* to generate the no-missing SNP dataset. A one-dimensional site frequency spectrum (1D-SFS) was constructed for each species using Python script ‘‘easySFS’’^1^. As the ancestral state was unknown, the folded SFS was used to record the frequency of the minor allele. Subsequently, 200 bootstraps were implemented to produce the median estimation and 95% CI. The result was finally visualized in R.

## Results

### DNA Sequencing and SNP Calling

Sequencing RAD-Tags from the six *Cycas* species yielded a total of 8,756,635,626 clean reads across the 152 individuals. The number of sequence reads per sample ranged from 36,889,920 to 93,193,638 (median = 57,557,792; Supplementary Table 3). The average amount of data varied between 7.67 Gbp for *C. taiwaniana* and 9.17 Gbp for *C. dolichophylla*, which accounts for the large genome size of *Cycas*. Applying a series of strict filtering criteria mentioned above including the genotyping call rate, depth of coverage thresholds, MAF, and heterozygosity efficiently removed poor-quality tags and artefactual SNPs resulting from paralogous tags or sequencing errors. The resulting dataset was characterized by a minimum of 70% genotype call rate for each species, with at least five species, a 0.5 heterozygosity and 2% MAF threshold. Finally, Dataset 1 was obtained with a total of 240,016 biallelic SNPs for phylogenetic inference and genetic diversity analyses. The mean coverage per locus across individuals varied between 2.6 and 44.7, with the median value being 10.7 (Supplementary Figure 2A), and the number of loci per individual ranged from 18,947 to 40,777 (median = 30,430; Supplementary Figure 2B).

Two further datasets were generated aiming for other analyses by applying alternative SNP-calling and filtering strategies. Dataset 2 was filtered to examine patterns of genetic structure and gene flow among species. To mitigate the potential effect of linkage disequilibrium, only one random SNP per locus was kept, and the resulting Dataset 2 had 33,629 SNPs. The no-missing SNP Dataset 3 aimed for demographic analysis was generated by downsampling samples and rerunning the populations program as above, and the number of SNPs obtained for each species is listed in Supplementary Table 4.

### Genetic Diversity and Pairwise Differentiation

**FIGURE 2:**
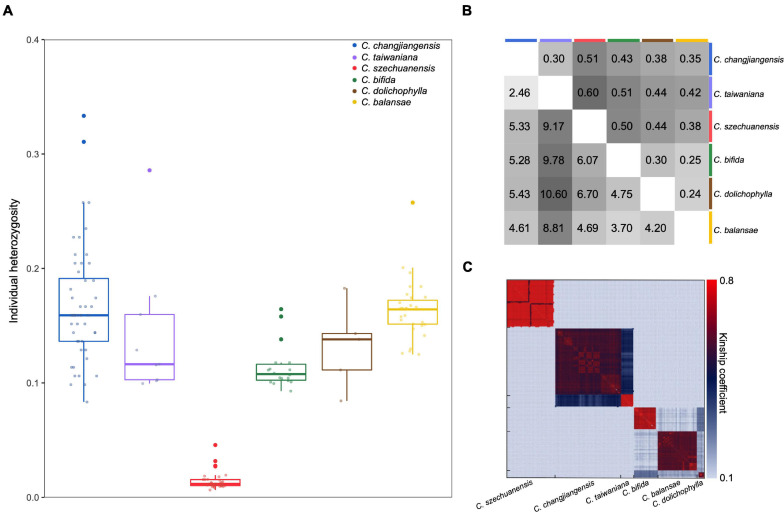
Genetic diversity and differentiation among the six *Cycas* species. **(A)** Individual heterozygosity for each species; **(B)** Pairwise genetic differentiation (*F*_*ST*_, upper triangular matrix) and pairwise sequence divergence (*D*_*XY*_, ×10^–3^, lower triangular matrix); and **(C)** Pairwise kinship coefficients among individuals within each species and between species.

Low levels of genetic diversity were consistently found across species, as evidenced by the expected heterozygosity and nucleotide diversity over all loci (*H*_*E*_ = 0.06–0.31, π = 0.00003–0.00262; Kruskal–Wallis test, *P* = 0.008; Table 1 and Figure 2A). Specifically, all individuals of *C. szechuanensis* showed extremely low heterozygosity, with values below 0.04 (Figure 2A). Overall, *C. szechuanensis* had extremely low levels of genetic diversity and high rates of inbreeding, while *C. dolichophylla* had a comparatively higher genetic diversity.

**TABLE 1:** Summary of genetic diversity statistics for the six studied species of *Cycas* based on 240,016 biallelic SNPs.

**Species**	***N***	***H*_*O*_**	***H*_*E*_**	**π (×10^–3^)**	***F*_*IS*_**
*C. changjiangensis*	52	0.17	0.19	0.12	0.14
*C. taiwaniana*	9	0.14	0.15	0.07	0.05
*C. szechuanensis*	37	0.01	0.06	0.03	0.83
*C. bifida*	18	0.11	0.13	0.33	0.10
*C. dolichophylla*	5	0.13	0.31	2.62	0.51
*C. balansae*	31	0.09	0.10	0.16	0.07

*N, the number of individuals analyzed; H_O_, observed heterozygosity; H_E_, expected heterozygosity; π, nucleotide diversity; and F_IS_, inbreeding coefficient.*

Pairwise *F*_*ST*_ values varied from 0.24 (*C. dolichophylla* vs. *C. balansae*) to 0.60 (*C. taiwaniana* vs. *C. szechuanensis*). Despite somewhat difference, pairwise *D*_*XY*_ showed generally congruent trend of species divergence, with the value ranging between 2.46 × 10^–3^ (*C. changjiangensis* vs. *C. taiwaniana*) to 1.06 × 10^–2^ per bp (*C. dolichophylla* vs. *C. taiwaniana*; Figure 2B). All of the comparisons were highly significant (*P* < 0.001), suggesting a high degree of genetic differentiation among the *Cycas* species. Pairwise kinship coefficients within species were significantly higher than those among species (Figure 2C). Additionally, *C. changjiangensis* and *C. taiwaniana* showed higher genomic kinship relatedness compared to other pairs of species (Figure 2C).

### Phylogenetic Analysis

**FIGURE 3:**
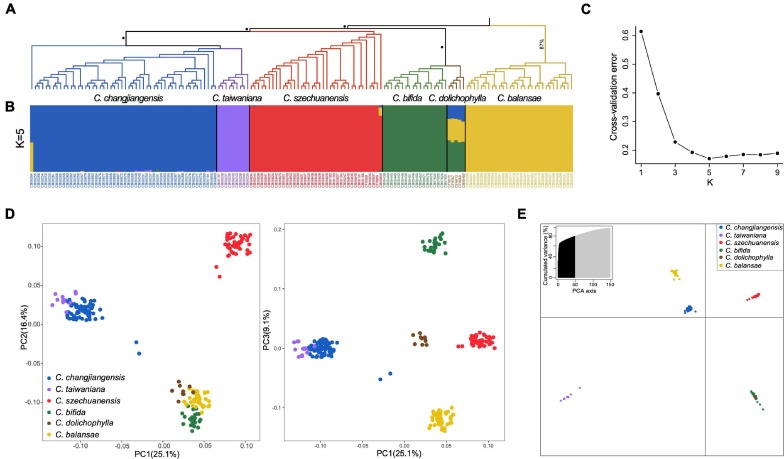
Phylogenetic relationship and estimated genetic structure for the six *Cycas* species. **(A)** Phylogenetic tree of the six *Cycas* species generated using IQ-Tree (Node with 100% bootstrap support is marked with a black dot); **(B)** Results from ADMIXTURE with *K* = 5 based on genome-wide SNP data; **(C)** Cross-validation plot for *K* = 1–9 obtained with ADMIXTURE; **(D)** Plots of the first three dimensions of principal component analysis for the six *Cycas* species, with the first three axes (PC1, PC2, and PC3) explaining 25.1, 16.4, and 9.1% of the variation, respectively; **(E)** Plot of the first two dimensions of discriminant analysis of principal component (DAPC), with the first two axes (PC1 and PC2) explaining 33.4 and 18.4% of the variation, respectively.

Both ML and SVDq methods yielded similar phylogenetic topologies with four main clades (Figure 3A and Supplementary Figure 3). Nearly all individuals of the same species clustered with high support, except one individual of *C. changjiangensis* (i.e., CB08284; Figure 3A). *C. dolichophylla* and *C. bifida* formed a clade. *C. szechuanensis* formed a single clade, which was sister to a clade consisting of *C. changjiangensis* and *C. taiwaniana*. Nearly all major nodes showed 100% bootstrap support in the ML tree, with the exception of the clade of *C. balansae* (87%; Figure 3A), whereas the clade of *C. dolichophylla* and *C. bifida* had a relatively low support value (74%) in the SVDq phylogenetic tree (Supplementary Figure 3), indicating potential ambiguity in this clade to some extent.

### Genetic Clustering

Analysis with ADMIXTURE inferred *K* = 5 as the optimal number of groups (Figure 3B). Individuals from each species were genetically assigned to a single group, while *C. dolichophylla* showed substantial admixture and shared an average of ∼45% of its genome with *C. bifida*, 34% with *C. balansae*, and 21% with *C. changjiangensis* (Figure 3B). On the other hand, statistical differences between *K* = 4 and *K* = 5 were relatively small according to cross-validation errors (Figure 3C). At *K* = 4, the supported groups were entirely congruent with genetic clusters generated with phylogenetic analysis (Supplementary Figure 4). Three major clusters (i.e., *C. szechuanensis*, *C. taiwaniana–C. changjiangensis*, and *C. balansae–C. dolichophylla–C. bifida*) were identified along the first two principal component (PC1 and PC2) axes in the PCA, while *C. balansae*, *C. dolichophylla*, and *C. bifida* were further separated along the PC3 axis, albeit with a much lower eigenvalue (Figure 3D). Additionally, five groups were also identified by the DAPC procedure, which was consistent with the results from ADMIXTURE (Figure 3E).

### Interspecific Gene Flow

**FIGURE 4:**
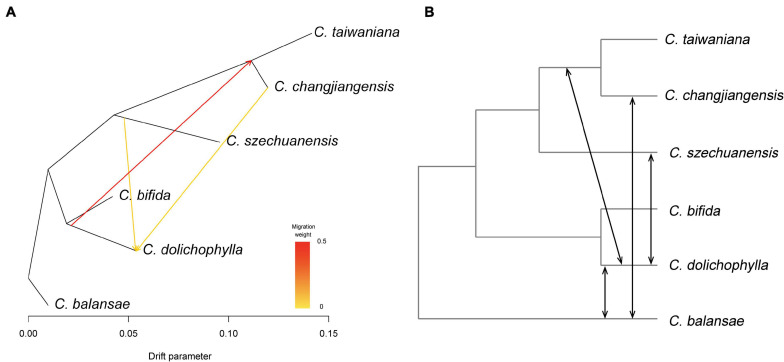
Hybridization among the six *Cycas* species. **(A)** Maximum likelihood tree inferred by Treemix, with the gene flow events depicted with arrows; **(B)** Proposed scenarios for interspecific gene flow revealed by *f*_4_-statistic and ABBA-BABA test.

The potential interspecific gene flow was examined with Treemix, *f*_4_-statistic, and ABBA-BABA test. A strong signal of gene flow was detected from *C. dolichophylla* to the common ancestor of *C. changjiangensis* and *C. taiwaniana* with Treemix (Figure 4A). Furthermore, two other relatively weak gene flow events were inferred from *C. szechuanensis* and *C. changjiangensis* to *C. dolichophylla* (Figure 4A). The *f*_4_-statistic was estimated for 12 four-taxon comparisons in total, with 10,880–21,332 biallelic SNPs that were extracted from our genomic dataset (Table 2). Most of the calculated *f*_4_ values (11 out of 12) were significantly different from zero, suggesting the occurrence of hybridization in *Cycas*. Introgression between *C. dolichophylla* or *C. balansae* and one of the two species *C. changjiangensis* or *C. taiwaniana*, was identified in our tests. In addition, the results from the *f*_4_-statistic could not preclude the presence of gene flow between *C. dolichophylla*, *C. balansae*, and *C. szechuanensis*. To further clarify whether *C. changjiangensis*, *C. taiwaniana*, or *C. szechuanensis* definitely involved the gene flow events mentioned above, ABBA-BABA test was performed. The results showed strong signals for gene flow between *C. changjiangensis*, *C. taiwaniana*, and *C. dolichophylla* (*P* < 0.01). However, the *D*-statistic was not significantly different from zero when the taxa combination was set as (((*C. taiwaniana*, *C. changjiangensis*), *C. dolichophylla*), O) (Table 3), indicating highly similar amounts of allele sharing between *C. dolichophylla* and both *C. taiwaniana* and *C. changjiangensis*. Gene exchange between *C. dolichophylla* and *C. szechuanensis* was also detected (Table 3). Only minimal gene flow was inferred between *C. dolichophylla*, *C. changjiangensis* and *C. balansae* (Table 3). The results obtained from both the *f*_4_-statistic and ABBA-BABA test were basically in agreement with our findings with Treemix (Figure 4B). Overall, complex patterns of gene flow were revealed in *Cycas*, with *C. dolichophylla* showing a relatively strong signal of genetic introgression.

**TABLE 2:** *f*_4_-statistic for the six *Cycas* species. *f*_4_-statistic was calculated in four-taxon comparisons to distinguish interspecific gene flow from incomplete lineage sorting (ILS).

**Taxon A**	**Taxon B**	**Taxon C**	**Taxon D**	**SNPs**	**%var**	***f*_4_**	***P***
*C. changjiangensis*	*C. taiwaniana*	*C. szechuanensis*	*C. bifida*	15,789	16.3	–0.00032	0.1890
*C. changjiangensis*	*C. taiwaniana*	*C. szechuanensis*	*C. dolichophylla*	16,623	16.4	–0.00146	**0.0010**
*C. changjiangensis*	*C. taiwaniana*	*C. szechuanensis*	*C. balansae*	16,350	19.5	–0.00097	**0.0000**
*C. changjiangensis*	*C. taiwaniana*	*C. bifida*	*C. dolichophylla*	11,249	13.2	–0.00084	**0.0000**
*C. changjiangensis*	*C. taiwaniana*	*C. bifida*	*C. balansae*	11,700	17.2	–0.00027	**0.0300**
*C. changjiangensis*	*C. taiwaniana*	*C. dolichophylla*	*C. balansae*	12,534	17.7	–0.00028	**0.0420**
*C. changjiangensis*	*C. szechuanensis*	*C. bifida*	*C. dolichophylla*	10,880	16.7	–0.00257	**0.0000**
*C. changjiangensis*	*C. szechuanensis*	*C. bifida*	*C. balansae*	11,331	22.2	0.01222	**0.0000**
*C. changjiangensis*	*C. szechuanensis*	*C. dolichophylla*	*C. balansae*	12,165	23.2	0.01605	**0.0000**
*C. taiwaniana*	*C. szechuanensis*	*C. bifida*	*C. dolichophylla*	20,047	11.3	–0.00315	**0.0000**
*C. taiwaniana*	*C. szechuanensis*	*C. bifida*	*C. balansae*	20,498	14.6	0.00968	**0.0000**
*C. taiwaniana*	*C. szechuanensis*	*C. dolichophylla*	*C. balansae*	21,332	15.0	0.01407	**0.0000**

*The number of biallelic SNPs used for each test and the percentage of SNPs that are variable in both pairs of sister species (%var) are given. Significance of *f*_4_ values was assessed with ILS-based simulations. Bold font indicates significance at *P* = 0.05.*

**TABLE 3:** ABBA-BABA analysis in the six species of *Cycas*.

**P1**	**P2**	**P3**	***D***	***P***
*C. bifida*	*C. dolichophylla*	*C. changjiangensis*	0.24	**<0.01**
*C. bifida*	*C. dolichophylla*	*C. taiwaniana*	0.25	**<0.01**
*C. bifida*	*C. dolichophylla*	*C. szechuanensis*	0.17	**<0.01**
*C. szechuanensis*	*C. taiwaniana*	*C. dolichophylla*	0.40	**<0.01**
*C. szechuanensis*	*C. changjiangensis*	*C. dolichophylla*	0.46	**<0.01**
*C. taiwaniana*	*C. changjiangensis*	*C. dolichophylla*	0.03	0.10
*C. changjiangensis*	*C. dolichophylla*	*C. balansae*	0.08	**<0.01**
*C. dolichophylla*	*C. bifida*	*C. balansae*	0.01	0.30
*C. taiwaniana*	*C. dolichophylla*	*C. balansae*	0.05	0.05
*C. szechuanensis*	*C. dolichophylla*	*C. balansae*	0.04	0.13
*C. szechuanensis*	*C. bifida*	*C. balansae*	0.05	0.09
*C. szechuanensis*	*C. changjiangensis*	*C. balansae*	0.06	**0.03**
*C. szechuanensis*	*C. taiwaniana*	*C. balansae*	0.03	0.15
*C. changjiangensis*	*C. taiwaniana*	*C. balansae*	0.01	0.43

*Bold font indicates significance at *P* = 0.05.*

### Changes in Population Size

**FIGURE 5:**
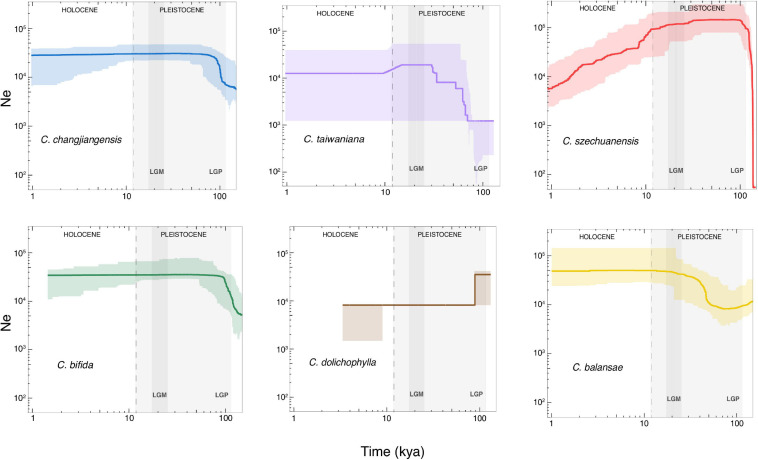
Demographic history inferred by Stairway Plot for the six studied species of *Cycas*. The *x* axis indicates time before present in thousand years ago (kya) on a log scale, and the *y* axis represents the effective population size. The bold color curve and the light color shaded areas show the median estimate and 95% confidence interval, respectively, based on 200 bootstrapped sequences for each species. The light and dark gray shaded areas indicate the last glacial period (LGP) and the Last Glacial Maximum (LGM), respectively.

The changes in effective population size dating from 1 to 100 thousand years ago (kya) were inferred for each species using Stairway Plot (Figure 5). *Cycas changjiangensis* and *C. bifida* shared similar population size change trajectories, where population expansion occurred right before the last glacial period (LGP) at ∼100 kya and lasted during the early LGP, followed by a relatively stable demography. *C szechuanensis* underwent a rapid decline since the Last Glacial Maximum (LGM) after a sharp expansion right before the LGP. Both *C. taiwaniana* and *C. balansae* experienced population expansions at ∼70 kya, continuing through LGP until the beginning of LGM. *Cycas taiwaniana* was detected with a further short-term slight population contraction right after the LGM at approximately 15 kya. *Cycas dolichophylla* experienced a drastic contraction at ∼70 kya, while the trajectory showed a strong stairstep pattern with a severely narrow CI.

## Discussion

### Efficiency of RADseq for Plants With Large Genomes

The availability of genome-wide datasets has become critical for understanding evolutionary information in the genomic era. However, performing population genomic analysis in non-model species with large genomes may be challenging due to the high costs and analytical complexity associated with the development of genome-wide markers (Parchman et al., 2018; Weisrock et al., 2018). RADseq permits quick sequencing with the advantage of drastic reduction in both cost and complexity, thus offering new avenues for phylogenetics and population genomics in such taxa. However, very few empirical studies using RADseq have been reported in plants with large and complex genomes (e.g., Clugston et al., 2019; Gargiulo et al., 2021). Gargiulo et al. (2021) proposed the necessity of bioinformatic filtering in RAD sequencing for non-model species with large genomes to obtain the true population genetic inference. More importantly, Clugston et al. (2019) demonstrated the applicability of RADseq across ten genera representing 13 species of Cycadales distributed in Australia (genomes up to approximately 60 Gbp). In the present study, tens and hundreds of thousands of SNPs were successfully obtained for genetic structure and phylogenetic analysis, respectively, across the six *Cycas* species distributed in South China. The strict quality checks and optimization of parameter sets could ensure the reliability of our data, and the robustness of data shown in our preliminary analysis highlighted their applicability in our subsequent analysis. Our study provides empirical evidence on the efficiency of RADseq for conservation genomic studies on species with large and complex genomes.

### Genetic Diversity, Phylogenetic Relationships, and Genetic Structure

Our analysis revealed consistently low genetic diversity (measured as *H*_*E*_ and π) and strong genetic differentiation across the six species of *Cycas* with a large number of genome-wide SNPs. Specifically, *C. szechuanensis* showed the lowest genetic diversity (*H*_*E*_ = 0.06), which was consistent with the findings from a previous study (Zheng et al., 2017). Our results support the arguments that plants with small populations, especially for rare species, generally have lower genetic diversity and higher genetic differentiation than those with large populations (Cole, 2003). Low intrapopulation genetic variation and relatively high genetic differentiation have been considered as biological and evolutionary characteristics of cycads (Walters and Decker-Walters, 1991), which has been demonstrated in other Asian inland cycads, such as *C. balansae* (Xiao and Gong, 2006), *C. simplicipinna* (Feng et al., 2014), *C. chenii* (Yang et al., 2016), *C. debaoensis* (Zhan et al., 2011), and *C. diannanensis* (Liu et al., 2015). However, some studies detected a high level of genetic diversity for several *Cycas* species in China based on microsatellite data, especially those with wide distribution ranges (Zheng et al., 2016; Feng X. et al., 2017; Xiao et al., 2020). Our results revealed significantly lower genetic diversity when compared with these congeneric studies, even for the same species, namely, *C. balansae* and *C. szechuanensis*. This difference must not be simply attributed to the sampling scheme, as *C. szechuanensis* only has a few cultivated individuals but no wild population recorded. Further, our study may provide more robust estimates of genetic diversity for such taxa with large genomes, given that the informative markers used are randomly scattered across the genome rather than a few loci. Low genetic diversity is more likely an inherent characteristic of *Cycas* with extremely small populations, which may be attributed to the combined effect of severe geographical isolation, genetic drift, and inbreeding as evidenced by the positive *F*_*IS*_ detected in our study.

Four main phylogenetic clades were recovered among the six *Cycas* species with both ML and SVDq methods. The largely monophyletic relationships of individuals within species suggested that species delimitations are basically coherent. *Cycas changjiangensis* and *C. bifida* were recovered as sisters to *C. taiwaniana* and *C. dolichophylla*, respectively, which is generally congruent with the topology of *Cycas* reconstructed using both plastid and nuclear loci (Mankga et al., 2020). PCA further demonstrated the close relationship between *C. changjiangensis* and *C. taiwaniana* and between *C. bifida* and *C. dolichophylla*. Recently, Feng et al. (2021) performed species delimitation on the *C. taiwaniana* complex based on genetic data of DNA sequences and microsatellites and proposed that *C. changjiangensis* and *C. taiwaniana* must be treated as one single species. However, both the ADMIXTURE and DAPC analyses in our study clearly suggested independent lineages between *C. changjiangensis* and *C. taiwaniana*, indicating that they belong to distinct evolutionary groups at the genomic level, despite their close relationship. Additionally, our results revealed independent clusters with a low admixture of genetic composition among the six *Cycas* species. While individuals of *C. dolichophylla* showed substantial genetic mixing in the analysis with ADMIXTURE (Figure 3B), which implies that hybridization might have played a role in the evolution of this species (but see below). Furthermore, individuals of *C. bifida* and *C. dolichophylla* always clustered together as a single genetic group for varying values of *K*, as demonstrated by DAPC, suggesting genetic introgression or ILS.

### Genomic Evidence of Hybridization in *Cycas*

Despite the high genetic differentiation detected among species, considerable genetic admixture was detected in *C. dolichophylla*, with substantial genome sharing with *C. bifida*, *C. balansae* and *C. changjiangensis*. The Treemix analysis identified bidirectional but asymmetrical gene flow between *C. dolichophylla* and *C. changjiangensis*. Relatively weak gene flow was also inferred from *C. szechuanensis* to *C. dolichophylla*. Comprehensive dated molecular phylogenies demonstrated recent rapid radiation of *Cycas* (Nagalingum et al., 2011; Mankga et al., 2020). Recently diverged species tend to be involved with incomplete reproductive barriers and may hybridize in sympatry (Fontaine et al., 2015). In contrast, ILS frequently occurs during rapid speciation, where ancestral polymorphisms may be randomly sorted in the descendant lineages. Hence, differentiating between signals of gene flow and ILS is especially important in understanding the evolutionary history of such recent rapid radiation species. Both *f*_4_-statistic and ABBA-BABA analysis have been demonstrated to be effective in discriminating genome-wide hybrid introgression from ILS (Reich et al., 2009; Green et al., 2010; Durand et al., 2011). Interestingly, our results from both *f*_4_-statistic and ABBA-BABA analysis generally aligned with the findings of Treemix and further provided evidence for substantial gene flow between *C. changjiangensis*, *C. taiwaniana*, and *C. dolichophylla* and between *C. dolichophylla* and *C. szechuanensis*, while only cases of minimal gene flow were inferred between *C. dolichophylla*, *C. changjiangensis* and *C. balansae*. Highly similar extents of allele sharing between *C. taiwaniana*, *C. changjiangensis*, and *C. dolichophylla* suggested that the taxon involved in genetic admixing with *C. dolichophylla* may be the ancestor of *C. taiwaniana* and *C. changjiangensis*, as detected with Treemix, indicating that ancient admixture occurs in *Cycas*. In contrast, the phenomenon of current hybridization has also been reported in cultivated populations. For example, bidirectional but asymmetric introgression was detected with amplified fragment length polymorphism markers between *C. revoluta* and *C. taitungensis*, as a result of the recently horticultural introduction of *C. revoluta* in eastern Taiwan (Chiang et al., 2013).

Geographic isolation contributes to genetic differentiation due to the inherently extremely small and isolated populations in *Cycas*. However, the lack of pollinator specificity, together with apparently weak inherent fertility barriers because of their recent radiation, may hasten the occurrence of hybridization for sympatric *Cycas* species. Morphologically intermediated individuals between *C. bifida*, *C. ferruginea*, and *C. dolichophylla* have been identified in nature (Vietnam), which is potentially indicative of natural hybridization in *Cycas* (Averyanov et al., 2014). More detailed studies must be performed to assess the spatial and temporal pattern of interspecific gene flow and their potential effects on the diversity and fitness of *Cycas*. Nevertheless, the relatively high genetic diversity in *C. dolichophylla* and *C. changjiangensis* detected in our study suggests that hybridization between divergent lineages may have contributed to diversity during the process of rapid radiation in *Cycas* to some extent. To the best of our knowledge, our study represents the first clear case of genomic-level natural hybridization in *Cycas*, thus providing a novel perspective on evolution in such recently radiated living fossil taxa.

### Complex Patterns of Demographic History

Estimating long-term demographic history is important to elucidate the genetic characteristics of species (Hewitt, 2000; Ekblom et al., 2018). The dynamics of demographic history inferred from genetic data provide useful information to understand how climate oscillation, habitat disturbance, and landscape evolvement affect population fitness and species viability (Selwood et al., 2015). Our results showed that all the six *Cycas* species underwent substantial *N*_*e*_ fluctuation with distinct trajectories during the late Pleistocene (∼100–11 kya), corresponding to the LGP interrupted by shorter interglacial intervals with warmer and moister climates (Comes and Kadereit, 1998). *C. szechuanensis* underwent a rapid and steady population decline since the LGM, indicating deep effects of climatic fluctuation on this species during both the late Pleistocene and Holocene. On the other hand, anthropogenic disturbances must not be ignored, as *C. szechuanensis* currently has only a few cultivated individuals with no wild populations reported (Zheng et al., 2017). Severe genetic loss may be induced by long-term population contraction, resulting in inbreeding depression and an extremely low level of genetic diversity (Arenas et al., 2012), which was detected in our study (*H*_*E*_ = 0.06). In addition, *C. dolichophylla* suffered from a sharp drop in the population size with a strong stairstep pattern during the early LGP (∼70 kya). Notably, the model was likely overfitted to the data as a result of the low sample size in *C. dolichophylla* (only five samples), as SFS-based demographic inference required at least ten samples per species (Patton et al., 2019). The rapid decline about 70 kya was also detected in *C. dolichophylla* based on nDNA (Zheng et al., 2016). Our results further demonstrated particular impacts of climate fluctuation during the late Pleistocene on the population dynamics of such species.

An unexpected finding of the present study was that nearly all of these species experienced population expansions, except *C. dolichophylla*. Especially for *C. bifida*, *C. balansae*, and *C. changjiangensis*, only the population expansion right before LGP occurred without subsequent population contraction. Our results seem to contradict the common findings from previous studies that population retreats occurred in southeast Asia, rather than expansion during Pleistocene glaciation (e.g., Gong et al., 2015; Liu et al., 2015; Feng et al., 2016; Zheng et al., 2016). However, distinct demographic dynamics have been detected in *Cycas* species distributed in South China. For example, *C. taitungensise* and *C. revoluta* were reported with population expansions during the last ice age (Chiang et al., 2009). More complex patterns of population dynamics were also revealed with different molecular markers in *Cycas* (e.g., *C. segmentifida*, Feng X. et al., 2017; *C. chenii*, Yang et al., 2016). Such contrasting responses to the Pleistocene climate oscillations could be attributed to distinct historical evolutionary processes of both topography and taxa (Huang et al., 2001; Gong et al., 2015). Specifically, range shift and adaptation during evolutionary history are central to plant species’ response to Quaternary climate change (Davis and Shaw, 2001). For example, *C. changjiangensis* is now endemic to Hainan, an island that was finally isolated from the mainland southern China after connection-disconnection oscillations during the last glacial period (Chang et al., 2012). During the LGM, Hainan was still connected with mainland southern China and northern Vietnam, with sea levels 80–100 m lower than at present (Huang et al., 1995). Presumably, *C. changjiangensis* retreated to the Hainan Island as a glacial refugium before it was finally isolated from the mainland. Similar demographic trajectories have also been detected in some other species distributed in Hainan (Peng et al., 2011; Chang et al., 2012). Further, *C. bifida* and *C. balansae* mainly occurred across the Red River Fault (RRF), a core distribution area for most species of *Cycas* in Asia (Xiao and Möller, 2015). Frequent geological activities, along with climate changes since the late Miocene in the RRF zone gave birth to complex topography and heterogeneous habitats (Zhu et al., 2009). The most recent dextral strike slip fault event occurred about 2.1 Mya (Xiang et al., 2007), which was generally in accordance with the time of *Cycas* colonizing South China (about 1.5 Mya; Mankga et al., 2020), although the exact origin of *Cycas* remains under debate (Xiao and Möller, 2015; Mankga et al., 2020). It is possible that the RRF played a key role as a refugium for such taxa during the Pleistocene glaciation period, which enabled them to survive through hostile climate oscillations between glacial and interglacial periods or even accumulate genetic diversity for subsequent persistence. In summary, our study uncovered complex patterns of demographic history in *Cycas* with extremely small populations, indicating that both climate fluctuation and frequent geological activities during the late Pleistocene exerted deep impacts on the population dynamics of such taxa in South China.

### Conservation Implications

Cycads are globally important relic plant groups, representing one of the most ancestral living seed plants. The naturally small population sizes and geographical isolation, together with severe human disturbance, make such taxa being among the most threatened plant groups. Genome-wide data could provide critical genetic information, thus are helpful in informing conservation decisions and designing management strategies regarding species of conservation concern. Our study demonstrated the promising applicability of RADseq in conservation genomic studies on plant taxa with large and complex genomes. Our results indicated that five of the six studied species experienced population expansions during the late Pleistocene and South China probably has acted as a critical refugium for most *Cycas* species in the area. Hence, *in situ* conservation, including habitat conservation, represents the most effective way to maximize genetic diversity for such taxa. However, low levels of genetic diversity and substantial inbreeding suggest inevitable loss of genetic variation to some extent. Both *ex situ* conservation and reintroduction are therefore urgently needed, especially for those species that could not be well preserved in their natural habitats due to severe human disturbance. The individuals harboring high genome-wide heterozygosity (Figure 2A) may be considered important resources for further germplasm collections. Considering the conflict between the limited capacity of *ex situ* conservation facilities and the increasing number of target species for conservation, it may be unavoidable to place different species of *Cycas* within the pollination range of each other, which makes it more difficult to maintain the genetic integrity of distinct species. Our study provides genomic evidence of interspecific gene flow in *Cycas*, together with the potential hybrids detected in nature (Averyanov et al., 2014) or in cultivated populations (Chiang et al., 2013), highlighting the need to evaluate the consequences of hybridization before performing large-scale *ex situ* conservation. Further, long-term monitoring programs must be designed to ensure the efficiency of both *in situ* and *ex situ* conservation for these species. Given the great significance of cycads as a radical transition in the evolution of plant biodiversity, our study provides important insights into the mechanisms of diversification in such recently radiated living fossil taxa.

## Data Availability Statement

The datasets presented in this study can be found in online repositories. The names of the repository/repositories and accession number(s) can be found below: https://www.ncbi.nlm.nih.gov/sra/PRJNA737036.

## Author Contributions

JW and YL conceived this work. BC performed the collection and identification of field materials. YT extracted DNA and analyzed the data. JW and YT wrote the manuscript. MK revised the manuscript. All authors contributed to the article and approved the submitted version.

## Conflict of Interest

The authors declare that the research was conducted in the absence of any commercial or financial relationships that could be construed as a potential conflict of interest.

## Publisher’s Note

All claims expressed in this article are solely those of the authors and do not necessarily represent those of their affiliated organizations, or those of the publisher, the editors and the reviewers. Any product that may be evaluated in this article, or claim that may be made by its manufacturer, is not guaranteed or endorsed by the publisher.

## Footnotes

1
https://github.com/isaacovercast/easySFS

## Supplementary Material

Supplementary Figure 1 PCA results for different parameter sets during preliminary analysis.

Supplementary Figure 2 Characteristics of SNPs for the Dataset 1 used in the present study. **(A)** Distribution of mean coverage per locus across 152 individuals for the genomic SNP of the six *Cycas* species; **(B)** Distribution of the number of loci per individual for the genomic SNP of the six *Cycas* species.

Supplementary Figure 3 Species tree reconstructed using SVDquartets algorithm, with posterior probabilities given at each node.

Supplementary Figure 4 Results from ADMIXTURE with *K* = 4 based on genome-wide SNP data.

Supplementary Table 1 Sampling information for each sample of the six studied *Cycas* species.

Supplementary Table 2 Number of SNPs generated with a series of parameter combinations (*m*, *M*, and *n*) during preliminary analysis with STACKS.

Supplementary Table 3 Sequence characteristics of RADseq for each sample of the six *Cycas* species.

Supplementary Table 4 SNP calling strategy, analysis module, and the number of SNPs obtained for each dataset used in the present study.
