# Supplementary figures and images for: Genome-Wide Evidence for Complex Hybridization and Demographic History in a Group of Cycas From China
Source: Front Genet. 2021 Aug 30;12:717200. doi: 10.3389/fgene.2021.717200 (PMC8435751; doi:10.3389/fgene.2021.717200)

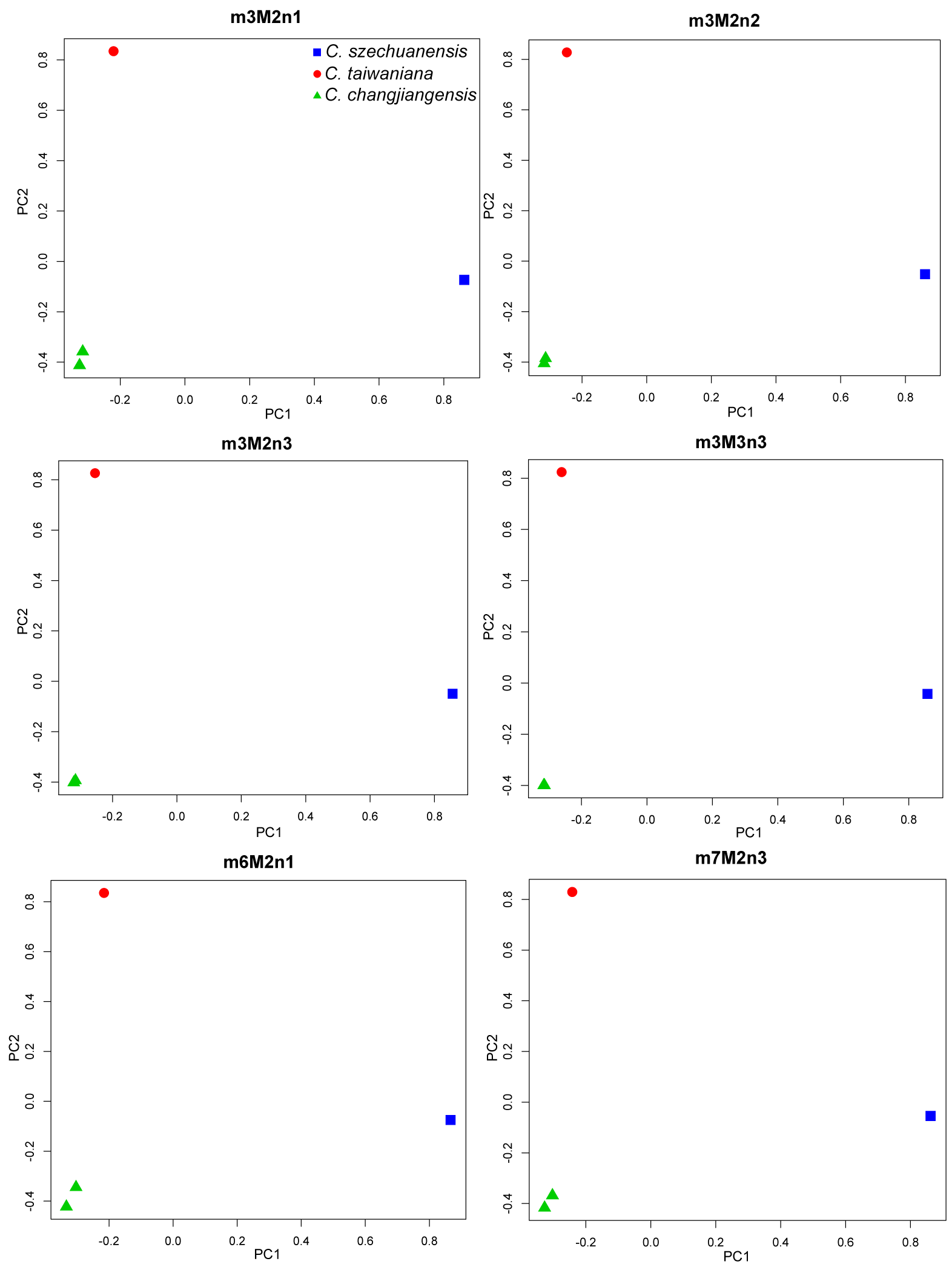

Supplement: Supplementary Figure 1 — PCA results for different parameter sets during preliminary analysis. [file Image_1.TIF]

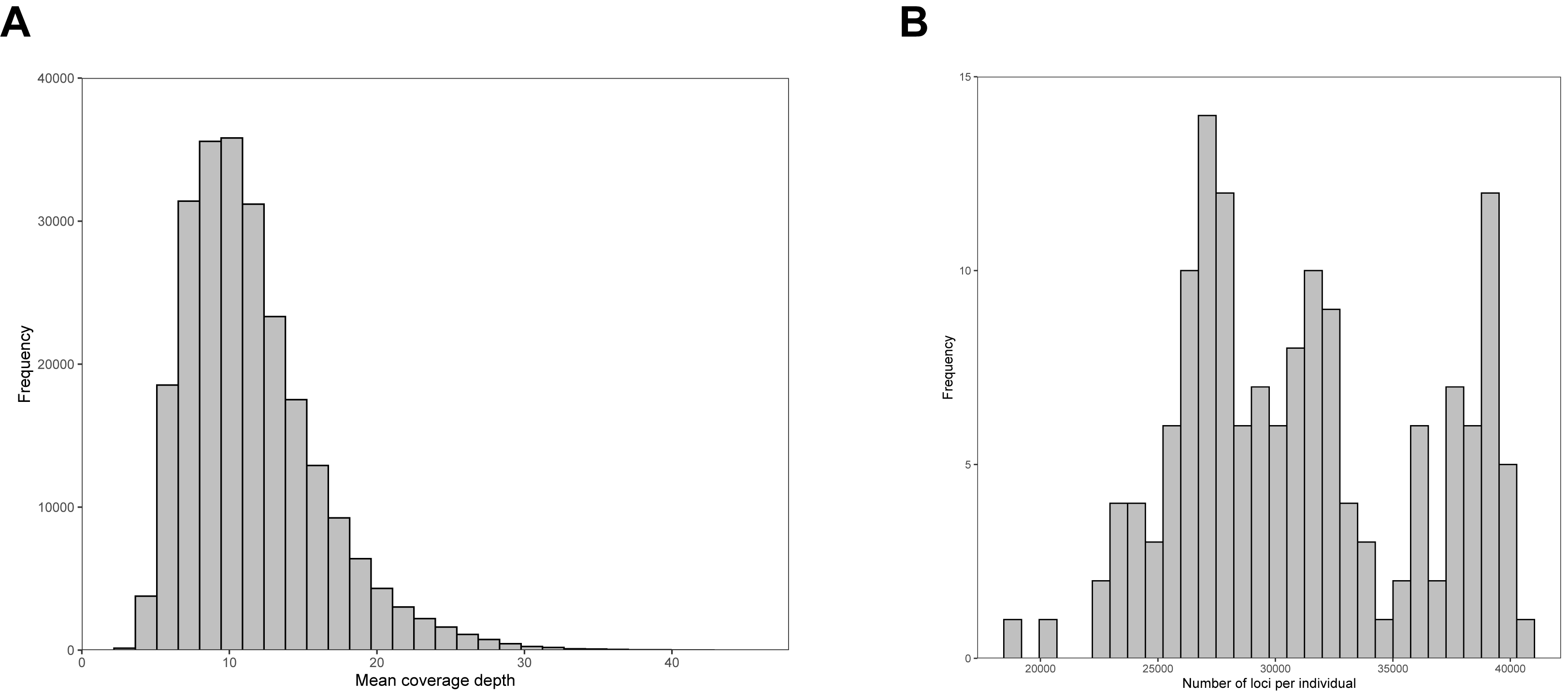

Supplement: Supplementary Figure 2 — Characteristics of SNPs for the Dataset 1 used in the present study. (A) Distribution of mean coverage per locus across 152 individuals for the genomic SNP of the six Cycas species; (B) Distribution of the number of loci per individual for the genomic SNP of the six Cycas species. [file Image_2.TIF]

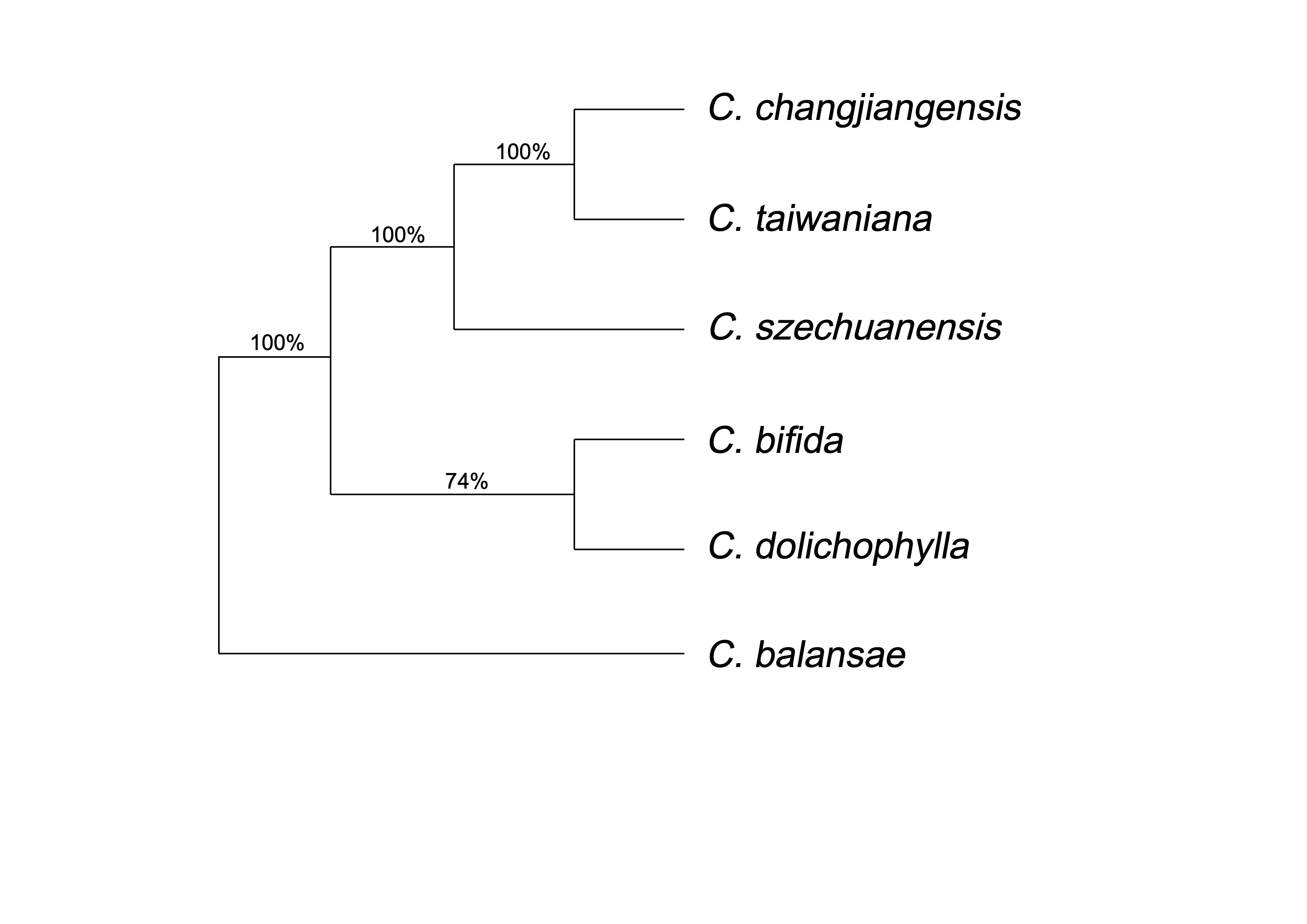

Supplement: Supplementary Figure 3 — Species tree reconstructed using SVDquartets algorithm, with posterior probabilities given at each node. [file Image_3.TIFF]

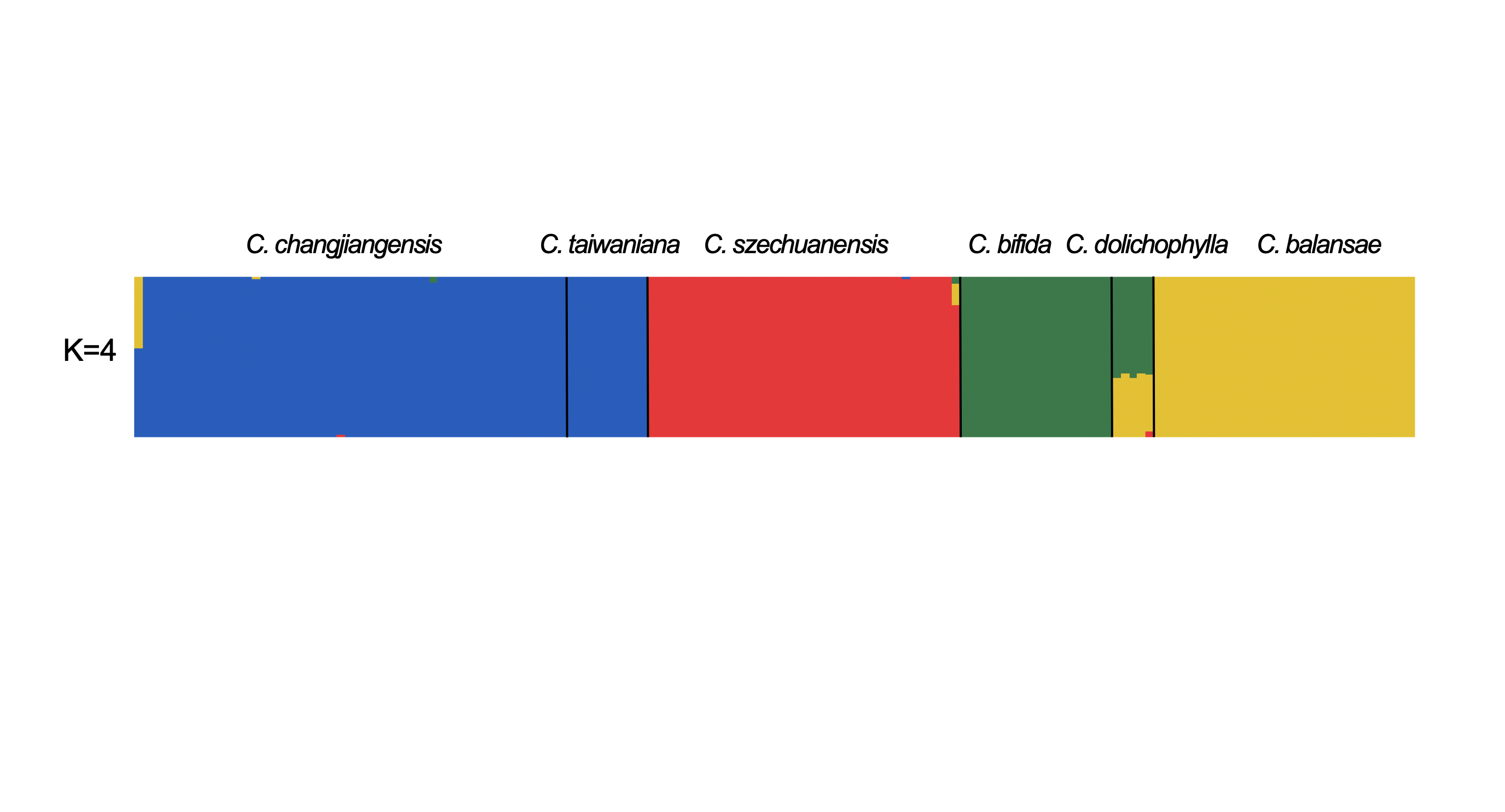

Supplement: Supplementary Figure 4 — Results from ADMIXTURE with K = 4 based on genome-wide SNP data. [file Image_4.TIFF]
